# Gaslini's tracheal team: preliminary experience after one year of paediatric airway reconstructive surgery

**DOI:** 10.1186/1824-7288-37-51

**Published:** 2011-10-26

**Authors:** Michele Torre, Marcello Carlucci, Stefano Avanzini, Vincenzo Jasonni, Philippe Monnier, Vincenzo Tarantino, Roberto D'Agostino, Renato Vallarino, Mirta Della Rocca, Andrea Moscatelli, Anna Dehò, Lucio Zannini, Nicola Stagnaro, Oliviero Sacco, Serena Panigada, Pietro Tuo

**Affiliations:** 1 Paediatric Surgery Unit, Gaslini Children's Hospital, Genova, Italy; 2 Otorhinolaryngology Unit, Centre Hospitalier Universitaire Vaudois, Lausanne, Switzerland; 3 Otorhinolaryngology Unit, Gaslini Children's Hospital, Genova, Italy; 4 Anaesthesia and Intensive Care Unit, Gaslini Children's Hospital, Genova, Italy; 5 Cardiology and Cardiac Surgery Unit, Gaslini Children's Hospital, Genova, Italy; 6 Service of Radiology, Gaslini Children's Hospital, Genova, Italy; 7 Pulmonary Diseases Unit, Gaslini Children's Hospital, Genova, Italy

## Abstract

**Background:**

congenital and acquired airway anomalies represent a relatively common albeit challenging problem in a national tertiary care hospital. In the past, most of these patients were sent to foreign Centres because of the lack of local experience in reconstructive surgery of the paediatric airway. In 2009, a dedicated team was established at our Institute. Gaslini's Tracheal Team includes different professionals, namely anaesthetists, intensive care specialists, neonatologists, pulmonologists, radiologists, and ENT, paediatric, and cardiovascular surgeons. The aim of this project was to provide these multidisciplinary patients, at any time, with intensive care, radiological investigations, diagnostic and operative endoscopy, reconstructive surgery, ECMO or cardiopulmonary bypass. Aim of this study is to present the results of the first year of airway reconstructive surgery activity of the Tracheal Team.

**Methods:**

between September 2009 and December 2010, 97 patients were evaluated or treated by our Gaslini Tracheal Team. Most of them were evaluated by both rigid and flexible endoscopy. In this study we included 8 patients who underwent reconstructive surgery of the airways. Four of them were referred to our centre or previously treated surgically or endoscopically without success in other Centres.

**Results:**

Eight patients required 9 surgical procedures on the airway: 4 cricotracheal resections, 2 laryngotracheoplasties, 1 tracheal resection, 1 repair of laryngeal cleft and 1 foreign body removal with cardiopulmonary bypass through anterior tracheal opening. Moreover, in 1 case secondary aortopexy was performed. All patients achieved finally good results, but two of them required two surgeries and most required endoscopic manoeuvres after surgery. The most complex cases were the ones who had already been previously treated.

**Conclusions:**

The treatment of paediatric airway anomalies requires a dedicated multidisciplinary approach and a single tertiary care Centre providing rapid access to endoscopic and surgical manoeuvres on upper and lower airways and the possibility to start immediately cardiopulmonary bypass or ECMO.

The preliminary experience of the Tracheal Team shows that good results can be obtained with this multidisciplinary approach in the treatment of complicated cases. The centralization of all the cases in one or few national Centres should be considered.

## Introduction

For many years the Gaslini Institute has been a reference Centre in Italy for the treatment of patients affected by complex malformations, extreme prematurity, complex syndromes, severe cardiac anomalies, and for the treatment of surgical patients referred by other Italian Hospitals. Congenital and acquired airway anomalies, though rare, represent in our Centre a relatively common event. They also represent a real challenge, because of the complexity of these patients, often requiring delicate diagnostic investigations and very demanding reconstructive treatments. The most frequent clinical presentations are foreign body inhalation, laryngomalacia, tracheomalacia and acquired subglottic stenosis (post intubation). Other congenital malformations involving the larynx (cleft, stenosis, atresia, membranes, cysts, angiomas) or the trachea (stenosis due to complete cartilaginous rings) are also observed. Each of these situations can represent a real challenge for the different specialists involved, namely anaesthetists, intensivists, neonatologists, pulmonologists, radiologists, ear-nose-throat (ENT) surgeons, paediatric surgeons and cardiovascular surgeons. In 2009, the "Tracheal Team" was established at Gaslini, including all the above-mentioned professional roles [a]. The aim was to avoid referring patients abroad for airway reconstructive surgery. To our knowledge our Institute is the only case in Italy in which all these specialists and dedicated facilities are present in a single Children Hospital. Aim of this study is to present the results of the first year of reconstructive surgery activity of the Tracheal Team.

## Patients and methods

Between September 2009 and December 2010, 97 patients were evaluated or treated by the Gaslini Tracheal Team. Most of these cases were studied by both flexible fiber optic bronchoscopy and rigid micro-laryngo-tracheo-bronchoscopy. Depending on the clinical picture, computerized tomography (CT), echocardiography, magnetic resonance (MR), or other diagnostic studies were performed in selected cases. Some patients presented with severe respiratory distress or were intubated. Besides patients admitted from the Emergency Department or the Intensive Care Unit for acute symptoms, others came from surgical units (Paediatric Surgery, Cardiovascular Surgery, ENT) or medical units (Pneumonology, High Dependency). About 30% of cases were transferred from other Hospitals. Most of the cases had an outpatient or day hospital evaluation. All the cases were jointly discussed by the Tracheal Team and data recorded prospectively and stored in a digital database according to the Personal Data Protection Act.

We included in the study patients who underwent reconstructive surgery of the affected airway. Patients who underwent tracheostomy, endoscopic treatment (with or without laser), endoscopic removal of foreign bodies, aortopexy because of tracheomalacia, surgical treatment of anomalous vascular ring compressing the airway and all the other treatments in which the trachea was not reconstructed, were not included in this study. Moreover, the patients treated before Tracheal Team setup in 2009 were also excluded from the study (two slide tracheoplasties for congenital tracheal stenosis, one laryngotracheal cleft repair and two laryngotracheoplasties). Clinical and instrumental data, type of operation, results and complications of the cases treated since September 2009 are reported.

**Figure 1:**
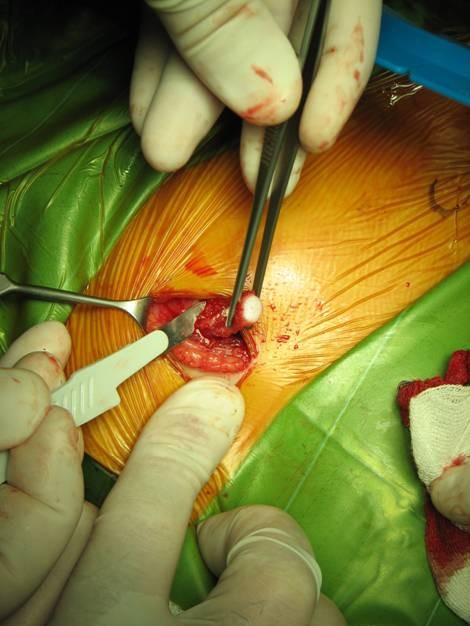
**LTP: costal cartilage graft collecting**.

**Figure 2:**
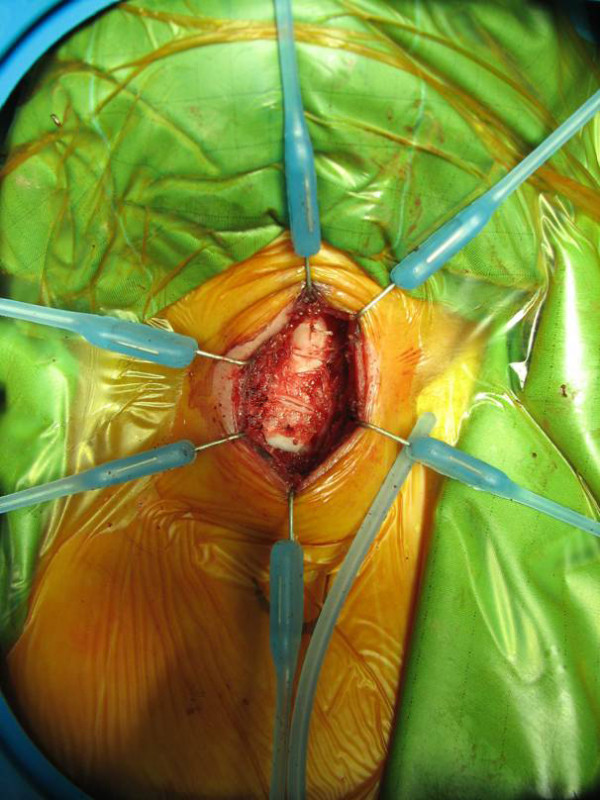
**LTP: the cartilage rib has enlarged the anterior wall of the airway**.

**Figure 3:**
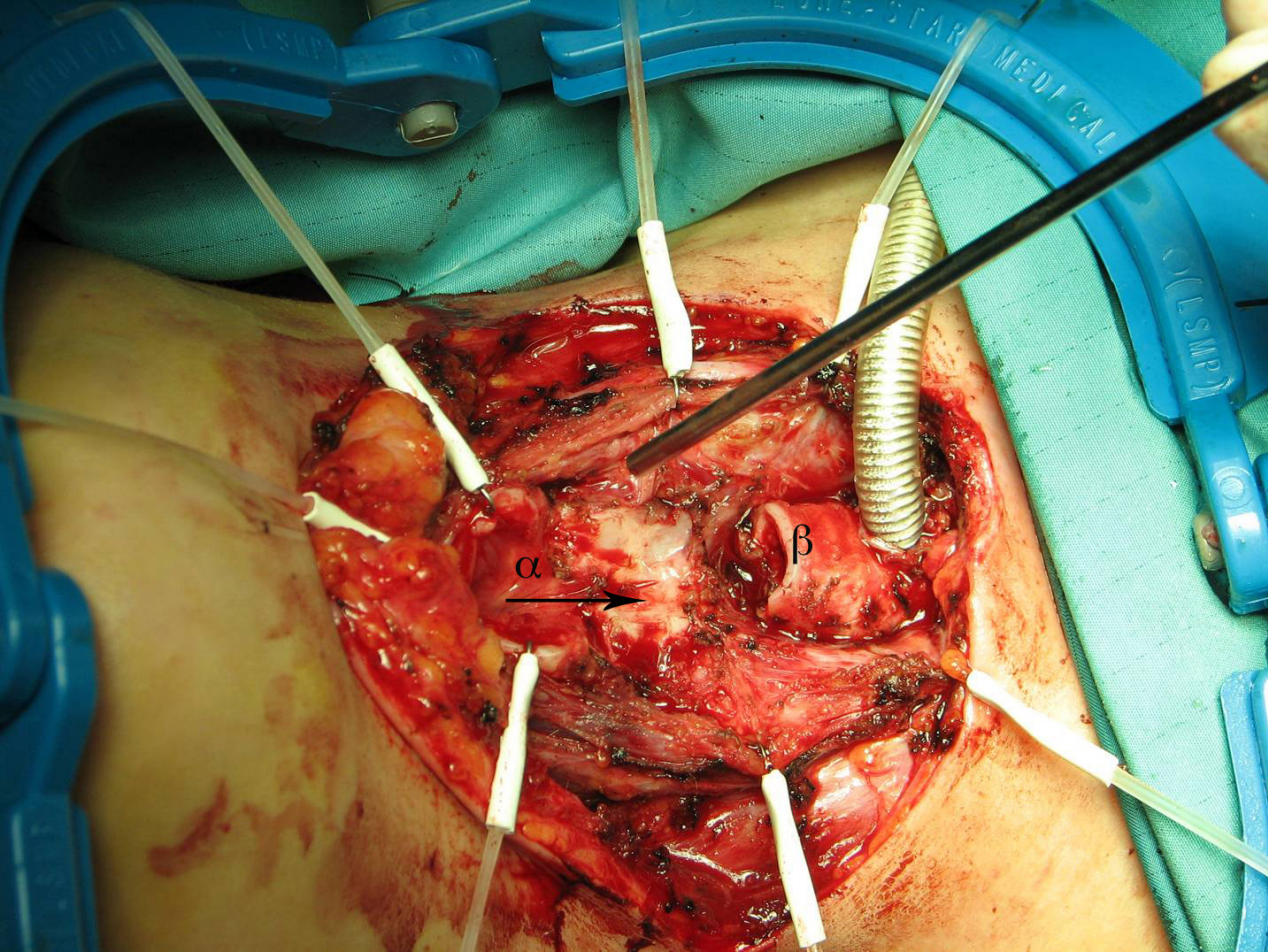
**CTR: the stenotic tract has been removed**. The opened thyroid cartilage (α), the anterior cricoid plate (arrow) and inferiorly the trachea (β) are shown.

**Figure 4:**
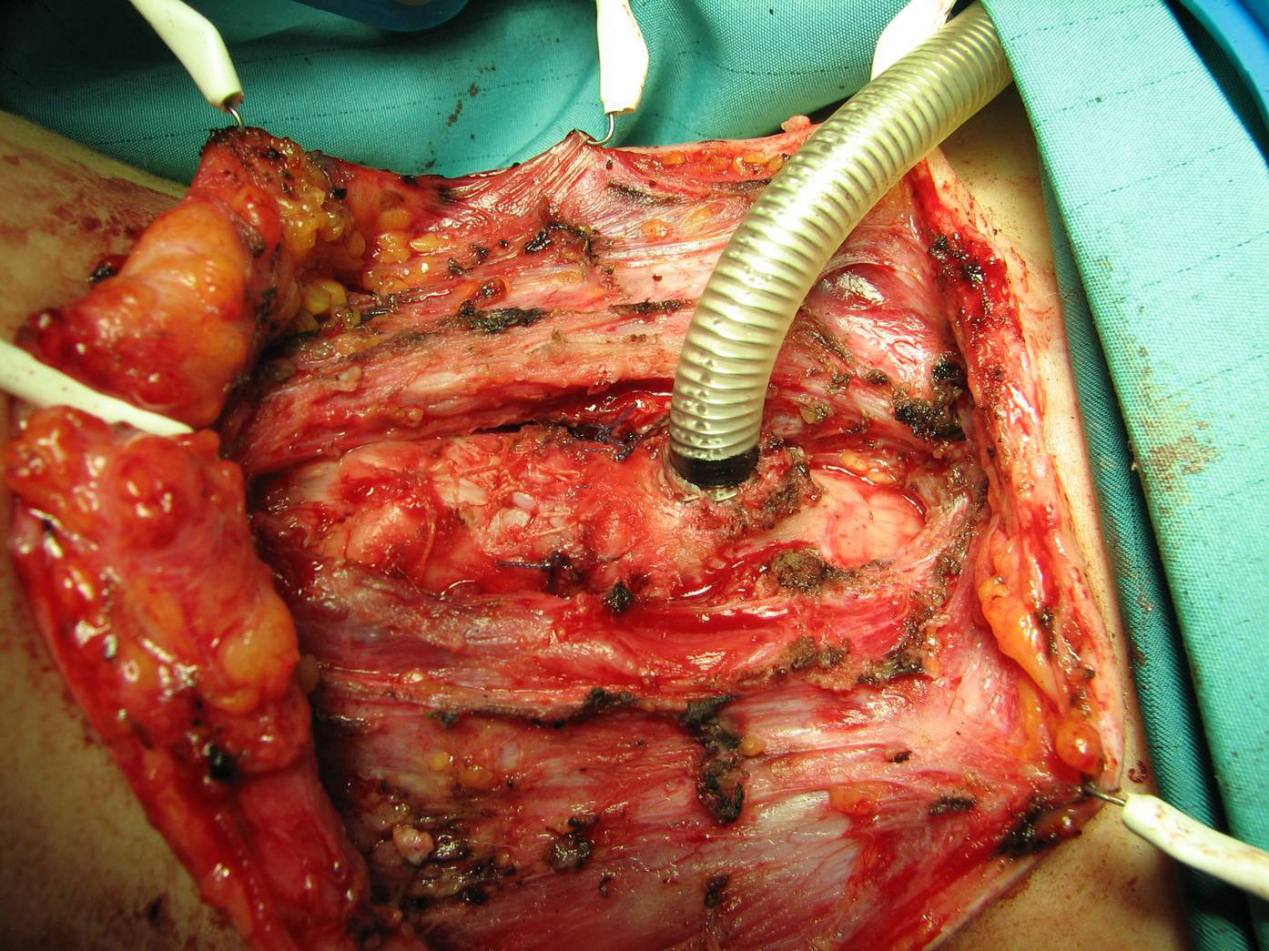
**CTR: Laryngo-tracheal anastomosis has been completed**. The cannula is still in situ below the anastomosis.

We adopted the Myer-Cotton classification of subglottic stenosis [1] into 4 degrees depending on the percentage of lumen occlusion (table 1). Laryngotracheoplasty (LTP) consists in the enlargement of the lumen of the airway by inserting a costal cartilage graft (usually in the anterior wall) (Figure 1 and 2). Cricotracheal resection (CTR) consists in the resection of the stenotic tract, including the anterior ring of the cricoid and the involved tracheal rings, and the anastomosis between the thyroid cartilage and the first unaffected tracheal ring (Figure 3 and 4). Indication for LTP was a grade 2 stenosis while indication for CTR was a grade 4 stenosis. Grade 3 stenoses were treated by LTP or CTR, depending on the surgeon's preference. Extended CTR was indicated in the treatment of transglottic stenosis which extends from above and below the glottis and involves the vocal chords. Extended CTR also included opening of all the thyroid cartilage in the midline and the insertion of a costal graft in the posterior wall to keep the glottis open.

**Table 1:** Myer-Cotton classification of subglottic stenosis

	Grade 1°	Grade 2°	Grade 3°	Grade 4°
% of lumen obstruction	< 50%	50-70%	70-99%	100%

## Results

Among the 97 patients evaluated by the Tracheal Team in the study period, 8 needed 9 surgical airway reconstruction procedures, namely 4 CTR, 2 LTP, 1 tracheal resection, 1 laryngeal cleft repair and 1 foreign body removal with cardiopulmonary bypass. In 1 case, secondary aortopexy was performed. All cases were investigated endoscopically before surgical treatment. The patients' clinical history is summarized in table 2.

**Table 2:** Patient's clinical history, management and follow-up

	Sex Age	Diagnosis	Tracheostomy pre-surgery	Type of Surgery	Tracheostomy maintenance post-surgery	Days of intubation	Following surgery	Decannulation	Outcome
**1**	F/7 years	Acquired subglottic stenosis grade 4°	+	CTR	+	3	Endoscopic removal of granuloma	+	Good

**2**	F/6 months	Congenital subglottic stenosis grade 2°	-	LTP	-	5	-	+	Good

**3**	M/5 years	Tracheal inflammatory myofibroblastic tumor	+	TR	-	2	Endoscopic removal of granuloma and tracheal dilatation	+	Good

**4**	F/13 years	Acquired trans-glottic stenosis grade 4°	+	ECTR	+	3	-	-	Patent airway*

**5**	M/5 months	Acquired subglottic stenosis grade 3°	+	LTP	-	Failed extubation	CTR and tracheal dilatation	+	Good

**6**	F/8 months	Congenital subglottic stenosis grade 3° and tracheomalacia	-	CTR	-	7	-	+	Good

**7**	F/10 months	Laryngo-tracheal cleft grade 3°, oesophageal atresia type 3	+	LTCC	-	0	Aortopexy	+	Cleft closed**

**8**	F/11 years	Foreign body inhalation	-	Open removal in CEC	-	3	Surgical drainage of subcutaneous infection	+	Good

(CTR, cricotracheal resection; LTP, laryngotracheoplasty; TR, tracheal resection; ECTR, extended cricotracheal resection; LTCC, laryngotracheal cleft closure; CEC: cardiopulmonary bypass). * After surgery a progressive but slow improvement of dysphonia and dysphagia was observed (the patient is now using a phonation valve and the nasogastric tube is still in situ). ** After surgery a recurrence of tracheo-oesophageal fistula was observed. Further surgery will be scheduled.

### Case 1

7-year-old female, coming from Romania, where tracheostomy had been performed for grade 4subglottic stenosis after intubation for severe infectious respiratory distress during acute leukaemia. The patient recovered from the acute leukaemia and underwent CTR in our Institute, maintaining the tracheostomy. The tube was removed on the 3^rd ^post-operative day. Post-operative endoscopy showed a good outcome of laryngo-tracheal tract reconstruction but also showed 2 partially occluding granulomas just above the tracheostomy that were removed endoscopically. The following control, after 1 month, showed a good airway patency and decannulation was successfully performed.

### Case 2

8-year-old female with a history of difficult intubation for neurosurgical operation (treatment of craniostenosis). Fiber optic laryngoscopy showed grade 2 congenital subglottic stenosis that was corrected by performing LTP with anterior costal graft insertion. The tracheal tube was removed on the 5^ft ^post-operative day. Post-operative endoscopy showed a small granuloma on the first tracheal ring that did not require any further treatment. Follow-up evaluations showed good patency of the airway and regression of respiratory symptoms.

### Case 3

**Figure 5:**
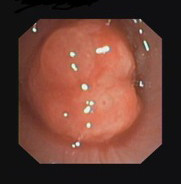
**Endoscopy: mass occluding 70% of the tracheal lumen**.

5-year-old asthmatic boy, followed by another Italian Institute, who was admitted to our unit for worsening dyspnoea. Endoscopy showed a mass occluding 70% of the tracheal lumen (Figure 5). Tracheostomy and biopsy were performed. Pathological results were compatible with inflammatory myofibroblastic tumor of the tracheal wall. Two weeks after tracheostomy, an extended CTR was performed, including the tracheostomy in the resection. The endotracheal tube was maintained for 2 days after surgery. Endoscopic follow-up showed one granuloma on the anastomosis, occluding 30% of tracheal lumen. The granuloma was removed endoscopically and the Bougie dilatation was performed. At the moment the patient is completely free of respiratory symptoms.

### Case 4

13-year-old girl, with history of extreme prematurity, affected by laryngo-tracheal stenosis subsequently to prolonged intubation, treated several times in many Centres (more than 20 endoscopic and surgical reconstructive procedures). She was admitted to our unit for persistent dysphonia, dysphagia and grade 4 transglottic stenosis. Extended CTR with a new tracheostomy were performed and endoluminal LT-Mold stent (Bredam S.A., St. Sulpice, Switzerland) was positioned. The endotracheal tube was removed on the 3^rd ^post-operative day. After 6 months the LT-Mold stent was removed by laryngoscopy that showed a patent airway widely. Phoniatric and logopedic follow-up showed a slow but progressive improvement of dysphonia (at present the patient is using a phonation valve) and dysphagia (although gastrostomy was required, which is still partially used).

### Case 5

Male newborn at the 28^th ^week of gestation; after surgical closure of persistent patent ductus arteriosus it was impossible to wean him from mechanical ventilation. Endoscopy showed post intubation grade 3 subglottic stenosis and paralysis of the left vocal chord. After several attempts at extubation, a tracheostomy was performed. At 5 months of age (weight 3.2 Kg) LTP with anterior costal graft insertion was performed and the tracheostomy was closed. The tracheal tube was removed on postoperative day 8. Because of respiratory distress, a new intubation was needed. Fiber optic bronchoscopy showed tracheomalacia at the site of previous tracheostomy. CTR was then performed. Three weeks postoperatively an endoscopic tracheal dilatation was performed, enlarging the lumen from 5 to 7 mm. Endoscopic follow-up after 3 months showed good tracheal patency and partially mobile left vocal chord. The patient is now completely symptom-free.

### Case 6

8-month-old girl, presenting stridor, tirage and dysphonia. Endoscopy showed tracheomalacia and congenital grade 3 subglottic stenosis, extending down to the 3^rd ^tracheal ring. She underwent CTR and anastomosis between thyroid cartilage and 4^th ^tracheal ring. After a first unsuccessful attempt of extubation 7 days after surgery, a 48 hours course of steroid therapy was given and the extubation was achieved. Endoscopic follow-up 2 months postoperatively showed paralysis of the right vocal chord, good appearance of the anastomosis, and good airway patency.

### Case 7

10-month-old girl transferred from another Italian Hospital, where she had been operated on for type 3 esophageal atresia, then re-operated for recurrent tracheo-esophageal fistula, and finally diagnosed with grade 3 laryngotracheal cleft that was treated endoscopically. Tracheobronchomalacia was diagnosed later. The patient came to us intubated and on mechanical ventilation that was difficult to maintain. Our endoscopic evaluation showed recurrence of the cleft in its distal end, at the first tracheal ring, and confirmed the diagnosis of severe tracheobronchomalacia. The endotracheal tube tended to move through the residual cleft into the esophagus, which explains the troubles with mechanical ventilation. A tracheostomy was performed providing a consistent improvement in ventilation parameters. Then she underwent two surgical procedures: the first was the three-layer closure of the recurrent cleft through an anterior trans-tracheal approach. Secondly, she underwent aortopexy through left anterior thoracotomy in order to improve the tracheomalacia. After surgery, the patient was weaned from mechanical ventilation and she is now on spontaneous ventilation. Although endoscopic evaluation showed a good closure of the cleft, it also showed recurrence of the tracheo-esophageal fistula divided at the time of surgery for esophageal atresia requiring a new correction through thoracotomy that will soon be scheduled.

### Case 8

11-year-old girl transferred from another European Hospital because of inhalation of a safety pin open a 75° angle. The sharp end of the pin was stuck in the mid tracheal wall with the tip at about 2 mm from the jugular/carotid axis. This position discouraged endoscopic removal, so open surgery was planned. Bronchoscopy-assisted tracheal intubation dislodged the pin towards the carina. The patient underwent emergency sternotomy and cardiopulmonary bypass was installed. The trachea was opened anteriorly in the midline and the pin removed. The anterior tracheal wall appeared slightly damaged by the pin, and was reinforced with a pericardial patch.

The patient was weaned from cardiopulmonary bypass without any problem, remained ventilated 3 days, and then was extubated. Subcutaneous infection at the surgical site required local drainage.

## Discussion

Paediatric airway congenital or acquired anomalies represent a challenge for many professionals, including intensivists, anaesthetists, neonatologists, paediatricians, respiratory physicians, ENT surgeons, paediatric surgeons, cardiac surgeons and radiologists. Previous experiences demonstrated that a multidisciplinary approach, which is possible in an Institution where all these professionals are available, gives the best results in terms of clinical outcome for the patients and cost effectiveness for the National Health System [2].

Our preliminary experience shows that good results can be achieved if a team of experts cooperates in a multidisciplinary way in a single tertiary Centre. The approach should be multidisciplinary during both diagnostic and therapeutic processes. In our opinion, both rigid and flexible endoscopy may be required for diagnostic procedures, as they are both necessary and complementary. Sometimes they have to be associated with gastro-esophageal endoscopy. Case 7 is a typical example in which the combination of rigid tracheoscopy and esophagoscopy allowed the diagnosis of the residual cleft, while flexible bronchoscopy showed tracheo-bronchomalacia. Regarding treatment, case 8 demonstrates that many services should be available in a single Centre for the treatment of highly demanding cases that require paediatric intensive care, rapid access to endoscopic and surgical manoeuvres on upper and lower airways and bronchi, possibility to start immediately cardiopulmonary bypass or ECMO. It is well known and reported in the literature that every endoscopic manoeuvre on the airways implies a risk of potential fatal complications, including acute respiratory insufficiency not manageable by mechanical ventilation and requiring immediate reconstructive surgery or ECMO [3]. Hence, endoscopic evaluation of challenging paediatric airways should ideally be performed only in Centres where a dedicated team is available at any time to face all possible emergencies, including ECMO or emergency sternotomy to reconstruct the airway using cardiopulmonary bypass.

A multidisciplinary team is also required for treatment, as frequently some intensive care or endoscopic procedures are required during the postoperative period. Our series showed that, in some cases, surgical repair alone is not sufficient to guarantee a good result. In particular, in our series of 8 patients, two reintubations, two granuloma resections and one endoscopic dilatation were required after the main surgical reconstruction.

In addiction, our series highlighted some aspects that need further discussion. Firstly, in patients already treated elsewhere without success or improperly, surgery is more demanding and may require multiple steps. For instance, patient 4 presented with a grade 4 transglottic stenosis following LTP and dozens of endoscopic dilatations performed in another Italian Centre. Moreover, in three internationally recognized Centres specialized in airway diseases (Marseille, Cincinnati and Lausanne), the patient was suggested three different options, namely contraindication to further treatment, LTP, and extended CTR, before treatment in our Institution, where CTR seemed to be an appropriate surgical solution. This is an emblematic case showing that choosing the correct treatment can be as difficult as the execution of treatment itself.

Secondly, CTR seems to offer better guarantees of success than LTP as treatment for grade 3 subglottic stenosis. This is confirmed by literature data [4] and by case 5, who underwent CTR after a partially successful LTP. Though more complex and technically demanding, CTR represents a more definitive solution for severe subglottic stenosis (grades 3 and 4), as the scar tissue is completely removed with re-approximation of the mucosa, and not only opened and enlarged, as in LTP. CTR can be performed also in low weight patients, as demonstrated in case 5 and in the literature [5,6]. If severe co-morbidities exist and/or vocal cord function is impaired, temporary tracheostomy could be suggested [7]. Otherwise, single stage CTR is performed (case 2 and 6). On the basis of our preliminary experience, whenever a tracheostomy has to be performed to allow ventilation, the site of the stoma can significantly influence the following CTR. In our opinion, tracheostomy site should be either close to the lower margin of the stenosis (on the 1^st ^or 2^nd ^tracheal ring), or far from it (5^th ^or 6^th ^tracheal ring), depending on whether CTR is performed in a single or double stage. If the tracheostomy is performed at an intermediate level (3^rd ^or 4^th ^tracheal ring), as suggested in many textbooks, the following airway reconstruction can be, as in our experience, much more complicated.

The last issue to be discussed is about the surgical options for laryngo-tracheal cleft. It is well known that grade 1 and 2 clefts can be successfully corrected endoscopically, and few cases of endoscopic treatment of grade 3 cleft have been reported [8,9]. However, the cleft relapsed in 50% of these cases [8], therefore we chose open surgical repair for our patient (case 7), who had already been treated and presented with relapsed cleft.

## Conclusions

Congenital or acquired paediatric airway anomalies require a multidisciplinary approach and a single Centre in which all dedicated professionals and services cooperate together, for both diagnosis and treatment.

The preliminary experience of the Tracheal Team shows that good results can be obtained in the treatment of complicated cases whenever an organized team including different professionals is set up in a single Centre.

Airway surgical reconstruction is only part of a multi-staged treatment, as other endoscopic procedures are often necessary to obtain a good result.

Those cases already treated previously without success are the most challenging.

The centralization of all cases in one or few national Centres has been reported in other Countries [2] as the optimal and most cost-effective solution to achieve the best outcome for these highly demanding patients.

## List of Abbreviations

CTR: cricotracheal resection; LTP: laryngotracheoplasty; ENT: ear-nose-throat; CT: Computerized Tomography; MR: magnetic resonance.

## Consent statement

Written informed consent was obtained from the patient for publication of this case report and accompanying images. A copy of the written consent is available for review by the Editor-in-Chief of this journal.

## Competing interests

The authors declare that they have no competing interests.

## Authors' contributions

All authors read and approved the final manuscript.

## Endnotes

[a] Tracheal Team members:

Specialist Clinical Nurses: Armanda Ferullo, Daniela Cordeglio, Daniela Tronconi

Anaesthetists/Intensivists/Neonatologists: Pietro Tuo, Renato Vallarino, Andrea Moscatelli, Mirta Della Rocca, Elisabetta Lampugnani

ENT Surgeons: Vincenzo Tarantino, Roberto D'Agostino, Philippe Monnier (External Consultant)

Paediatric Surgeons: Vincenzo Jasonni, Michele Torre, Stefano Avanzini, Marcello Carlucci

Cardiac Surgeons: Lucio Zannini, Francesco Santoro

Pulmonologists: Oliviero Sacco, Serena Panigada, Nicola Ullmann

Radiologists: Gianmichele Magnano, Nicola Stagnaro

Cardiologist: Maurizio Marasini

Psychologist: Giovanna Lenci

Speech and Language Therapist: Daniela Roncallo
